# Steroid Concentrations in Plasma, Whole Blood and Brain: Effects of Saline Perfusion to Remove Blood Contamination from Brain

**DOI:** 10.1371/journal.pone.0015727

**Published:** 2010-12-29

**Authors:** Matthew D. Taves, Kim L. Schmidt, Ilan M. Ruhr, Katarzyna Kapusta, Nora H. Prior, Kiran K. Soma

**Affiliations:** 1 Department of Psychology, University of British Columbia, Vancouver, Canada; 2 Department of Zoology, University of British Columbia, Vancouver, Canada; 3 Graduate Program in Neuroscience, University of British Columbia, Vancouver, Canada; 4 Department of Life Sciences, University of Strasbourg, Strasbourg, France; University of Alabama, United States of America

## Abstract

The brain and other organs locally synthesize steroids. Local synthesis is suggested when steroid levels are higher in tissue than in the circulation. However, measurement of both circulating and tissue steroid levels are subject to methodological considerations. For example, plasma samples are commonly used to estimate circulating steroid levels in whole blood, but steroid levels in plasma and whole blood could differ. In addition, tissue steroid measurements might be affected by blood contamination, which can be addressed experimentally by using saline perfusion to remove blood. In Study 1, we measured corticosterone and testosterone (T) levels in zebra finch *(Taeniopygia guttata)* plasma, whole blood, and red blood cells (RBC). We also compared corticosterone in plasma, whole blood, and RBC at baseline and after 60 min restraint stress. In Study 2, we quantified corticosterone, dehydroepiandrosterone (DHEA), T, and 17β-estradiol (E_2_) levels in the brains of sham-perfused or saline-perfused subjects. In Study 1, corticosterone and T concentrations were highest in plasma, significantly lower in whole blood, and lowest in RBC. In Study 2, saline perfusion unexpectedly increased corticosterone levels in the rostral telencephalon but not other regions. In contrast, saline perfusion decreased DHEA levels in caudal telencephalon and diencephalon. Saline perfusion also increased E_2_ levels in caudal telencephalon. In summary, when comparing local and systemic steroid levels, the inclusion of whole blood samples should prove useful. Moreover, blood contamination has little or no effect on measurement of brain steroid levels, suggesting that saline perfusion is not necessary prior to brain collection. Indeed, saline perfusion itself may elevate and lower steroid concentrations in a rapid, region-specific manner.

## Introduction

Endocrine research on steroids has traditionally focused on systemic levels in the general circulation. Many studies, however, have demonstrated that various organs synthesize steroids locally, and that these steroids often act locally without secretion into the blood [1]. Glucocorticoids, for example, are produced at extra-adrenal sites, including immune organs [2], [3], skin [4], and brain [5]. Sex steroids are locally produced in the brain, where they may act rapidly as neurotransmitters or neuromodulators in a wide variety of vertebrates [6], [7].

One type of evidence for local steroid synthesis is the presence of higher steroid concentrations in the organ of interest than in the circulation. Note that high local steroid levels might also reflect high receptor density, so other types of evidence are necessary. Nonetheless, comparing local and systemic steroid levels is useful and important [8]. Measurements of both circulating and tissue steroid levels are subject to methodological considerations that may affect the ability to detect high local steroid levels. For example, circulating steroid levels in blood are usually estimated using plasma (or serum) samples. However, steroid levels in plasma and whole blood can differ, as seen in a study of humans [9]. Assaying steroids in plasma may concentrate samples such that they no longer reflect what the tissues are actually bathed in and have access to. As a second example, tissue steroid measurements may be affected by the blood content of the tissue. Potential effects of blood contamination might be particularly problematic in well-vascularized tissue such as brain. Blood contamination can be removed via exsanguination, for example by transcardial perfusion with saline. Studies have compared saline-perfused and non-perfused rat [10] and mouse [11] brain for 17β-estradiol (E_2_) and corticosterone levels, respectively, and found no differences. These rodent studies suggest that blood contamination is not a major concern when measuring brain steroid levels, but such studies have not examined songbirds, an important model for neurosteroid research [12], [13].

Songbirds have been useful for investigating systemic and brain steroid levels [14]. Here, we used the zebra finch (*Taeniopygia guttata*) to compare circulating steroid levels in plasma and whole blood, and to determine whether blood contamination affects measurement of steroid levels in brain. In Study 1, we quantified corticosterone and testosterone (T) levels in plasma, whole blood, and red blood cells (RBC). Corticosterone levels were also measured at baseline and after restraint stress. Since plasma proteins such as corticosteroid-binding globulin (CBG) and albumin bind a large fraction of circulating steroids [9], [15], [16], we predicted that steroid concentrations would be high in plasma, intermediate in whole blood, and low in RBC (when equal volumes of each sample type were assayed). In Study 2, we quantified corticosterone, dehydroepiandrosterone (DHEA), T, and E_2_ levels in the brains of sham-perfused or saline-perfused subjects. We predicted that if blood contamination were a major problem, then saline perfusion would cause a global decrease in all brain steroid levels. In sham-perfused subjects, we also compared steroid concentrations in plasma, whole blood, and brain. We found that steroid concentrations were higher in plasma than in whole blood (Study 1), and that saline perfusion altered brain steroid concentrations in a region-specific manner that suggested that blood contamination of brain tissue was not a major problem (Study 2).

## Materials and Methods

### Subjects

Samples were collected from adult male zebra finches housed in a colony at the University of British Columbia. Birds were held under a 14∶10 light:dark cycle (lights on at 0800 h), with free access to food and water. Protocols were approved by the UBC Animal Care Committee (ID A07-0787) and conformed to the regulations established by the Canadian Council on Animal Care.

### Study 1: Steroid concentrations in plasma, whole blood, and red blood cells

In Study 1A, we examined corticosterone and T levels in plasma, whole blood and RBC (n = 12 subjects for corticosterone, 6 separate subjects for T). Blood samples (∼150 µl) were collected from the brachial vein using three heparinized microhematocrit tubes. Samples were obtained within 1.52 to 5.57 min (mean ± SEM  = 2.79±0.28 min) of initial disturbance. The blood in one tube was used to measure steroids in whole blood, and blood in the other two tubes was pooled and centrifuged to separate plasma and RBC. Blood was centrifuged for 10 min at 13,500 g, after which plasma was removed. Some plasma was likely still present in the RBC pellet [17]. Whole blood, plasma, and RBC (∼50 µl each) were stored at −20°C. All subjects were sampled between 0800 and 0900 h to control for possible diel variation in steroid levels.

In Study 1B, we examined baseline and stressed levels of corticosterone in plasma, whole blood, and RBC. Blood samples were collected from the brachial vein. Baseline samples (n = 6) were all obtained within 3 min (2.20±0.25 min); samples collected in this time have baseline corticosterone concentrations [18]. Stressed samples were obtained from separate individuals (n = 5) after 60 min (63.43±0.59 min) of restraint in an opaque cloth bag. The same subjects could not be bled at both baseline and after 60 min, because of the quantity of blood required and the small size of zebra finches. In zebra finches, plasma CBG levels decrease after 60 min of restraint [15], which might affect the compartmentalization of corticosterone between plasma and RBC. All subjects were sampled between 0800 and 0930 h.

For Studies 1A and 1B, steroid concentrations are given in ng/g, as RBC samples were weighed to determine the amount of tissue. For plasma and whole blood samples, ng/g measurements are nearly equivalent to ng/ml ([2] and unpub. results). For each subject, the amounts of plasma, whole blood, and RBC extracted and assayed were the same, except for two stressed subjects in Study 1B. For these two subjects, smaller (but equal) amounts of plasma and whole blood were used, since the steroid values were greater than the maximum point on the standard curve in an initial attempt.

### Study 2: Effects of saline perfusion on brain steroid levels

Subjects in Study 2 were kept under identical conditions as those in Study 1. Baseline blood samples were collected from the brachial vein within 3 min of initial disturbance (2.28±0.12 min). Animals were then rapidly anaesthetized with isoflurane, and the heart was quickly exposed. A butterfly needle was inserted into the left ventricle, and an incision was made in the right atrium. Then 10 ml of ice-cold heparinized isotonic avian saline (0.75% NaCl, 1 U/ml heparin) [19], [20] was used for saline perfusion (perfusion rate was ∼2.5 ml/min) (n = 11 subjects total). The perfusate exiting the right atrium was clear at the completion of saline perfusion. For sham-perfused subjects (n = 9), animals were similarly anaesthetized with isoflurane, and the heart was quickly exposed. The butterfly needle was held beside the heart as 10 ml of saline was expelled into the thoracic cavity. Cardiac blood was collected from sham-perfused birds at the end of the sham perfusion, using a heparinized syringe. Sham perfusions and saline perfusions were completed in similar amounts of time (sham perfusion: 10.48±0.40 min, saline perfusion: 11.11±0.31 min from initial disturbance; t_12_ = 1.07, p = 0.31). Note that the perfusion itself (from initiation to completion of saline expulsion) took ∼4 min. Birds were then rapidly decapitated, and brains were briefly chilled on wet ice before removal from the cranium. Sham-perfused brains (n = 9) were dark pink upon visual inspection. Of the saline-perfused brains (n = 11 total), six brains were completely pink or partially pink upon visual inspection, and five brains were completely white. Only the five completely white brains were analyzed, resulting in n = 5 saline-perfused brains. Right and left sides of the telencephalon were each bisected into rostral and caudal halves. Next, the diencephalon and cerebellum were collected (these regions were not split into right and left). Diencephalon was dissected to the depth of the anterior commissure. Dissected regions were rapidly frozen on dry ice and stored at −80°C.

### Steroid extraction and measurement

Steroids were extracted from all samples (plasma, whole blood, RBC, brain) in an identical manner, using solid phase extraction with C_18_ columns as previously described [21], [2], [14]. Briefly, we homogenized samples in ice-cold deionized water and then added HPLC-grade methanol. The following day, we centrifuged the samples, collected supernatants (up to 1 ml), and added 10 ml of deionized water to supernatants before loading onto C_18_ columns. Prior to sample loading, the columns had been primed with 3 ml HPLC-grade ethanol and equilibrated with deionized water (5 ml ×2). After sample loading, samples were washed with deionized water (5 ml ×2) for measurement of corticosterone and DHEA. For extraction of T and E_2_, we made a slight modification of the previous protocol and samples were washed with 40% HPLC-grade methanol in water (5 ml ×2), rather than water alone [22], [23]. In our preliminary studies, washing samples with 40% methanol gave higher recoveries for T and E_2_, and removed more interfering substances (presumably brain lipids). After sample washing, steroids were eluted with 90% HPLC-grade methanol (5 ml), and dried at 40°C in a vacuum centrifuge (ThermoElectron SPD111V Speedvac).

Steroids were measured in duplicate using specific and sensitive radioimmunoassays (RIAs) according to [24]–[27]. Recovery was assessed by spiking plasma, whole blood, RBC, and brain with known amounts of steroids and comparing these samples with unspiked samples from the same pool. Values were corrected for recovery. RIA details and recoveries for each steroid are given in **Table 1**. These assays have been validated for use with songbird plasma and tissue [21], [28], [27], [2], [29], [14], [16]. Rostral and caudal telencephalon samples were assayed for corticosterone and DHEA (right or left – randomly selected [14]). Diencephalon and cerebellum samples (not bisected into right and left halves) were also assayed for corticosterone and DHEA. We chose to measure corticosterone and DHEA as these steroids have been shown to have opposing effects on adult songbird neuroplasticity [28]. The remaining caudal telencephalon samples were assayed for both T and E_2_. The remaining rostral telencephalon samples were used in pilot studies to estimate hemoglobin content.

**Table 1:** Radioimmunoassay specifications.

Steroid	RIA kit	Modification	Detection limit (pg/tube)	Recovery (%)			
				Plasma	Whole Blood	RBC	Brain
Corticosterone	MP Biomed., cat.07120103	[24]	3.125	98.8	95.2	91.8	97.1
DHEA	Beckman, DSL-8900	[25]	2.0	92.4	94.8	n/a	102.1
Testosterone	Beckman, DSL-4100	[26]	0.44	84.9	80.1	93.1	98.0
17β–estradiol	Beckman, DSL-4800	[27]	0.188	79.8	n/a	n/a	89.7

Note. n/a  =  not assessed in these tissue types.

For measurement of corticosterone and DHEA, dried eluates were resuspended in 250 µl of PBSG (phosphate-buffered saline containing 0.1% gelatin) with 5% absolute ethanol, and 50 µl was transferred into each duplicate corticosterone RIA tube. The remaining 150 µl was brought up to 230 µl with PBSG, and 100 µl was transferred to each duplicate DHEA RIA tube. Thus 40% of the sample was used to assay corticosterone, and 52% of the sample was used to assay DHEA. For measurement of T and E_2_, dried eluates were resuspended in 1200 µl PBSG with 0.5% absolute ethanol, and 300 µl was transferred into each duplicate E_2_ RIA tube. The remaining 600 µl of resuspension was brought up to 1000 µl with PBSG, and 400 µl was transferred into each duplicate T RIA tube. Thus 50% of the sample was used to assay E_2_, and 40% was used to assay T.

### Statistical analysis

Nondetectable samples (those below the lowest standard on the standard curve) were set to zero [14]. Sample sizes vary in some cases, where there was insufficient sample to measure multiple steroids (e.g., some blood samples were greater than others, and could be assayed for more steroids). Data were analyzed using SPSS (version 11) and G*Power 3 [30] on an Apple computer. Data were tested using the Shapiro-Wilk test for normality and the Brown-Forsythe test for homogeneity of variance, and data were transformed when necessary.

In Study 1A, steroid distribution across blood compartments was analyzed with a one-way repeated-measures ANOVA. In Study 1B, data were analyzed with a two-way mixed-model ANOVA, with sample type (plasma, whole blood, RBC) as a within-subjects factor and with time (baseline, 60 min) as a between-subjects factor.

In Study 2, steroid concentrations in plasma and whole blood at baseline and after sham perfusion were analyzed with paired t-tests, because missing data precluded the use of a repeated-measures ANOVA. Steroid concentrations in each brain region were compared between groups using unpaired t-tests. Steroid concentrations in plasma, whole blood, and brain were compared using paired t-tests because of missing data. Effect sizes are presented as Cohen's *d* and Cohen's *f* for t-tests and ANOVAs, respectively [31]. Effect sizes for paired data were calculated using unpaired test statistics, as paired test statistics result in overestimation of effect size [32]. Tests were two-tailed and significance set at α = 0.05. Data are shown as mean ± SEM.

## Results

### Study 1: Steroid concentrations in plasma, whole blood, and red blood cells

**Figure 1:**
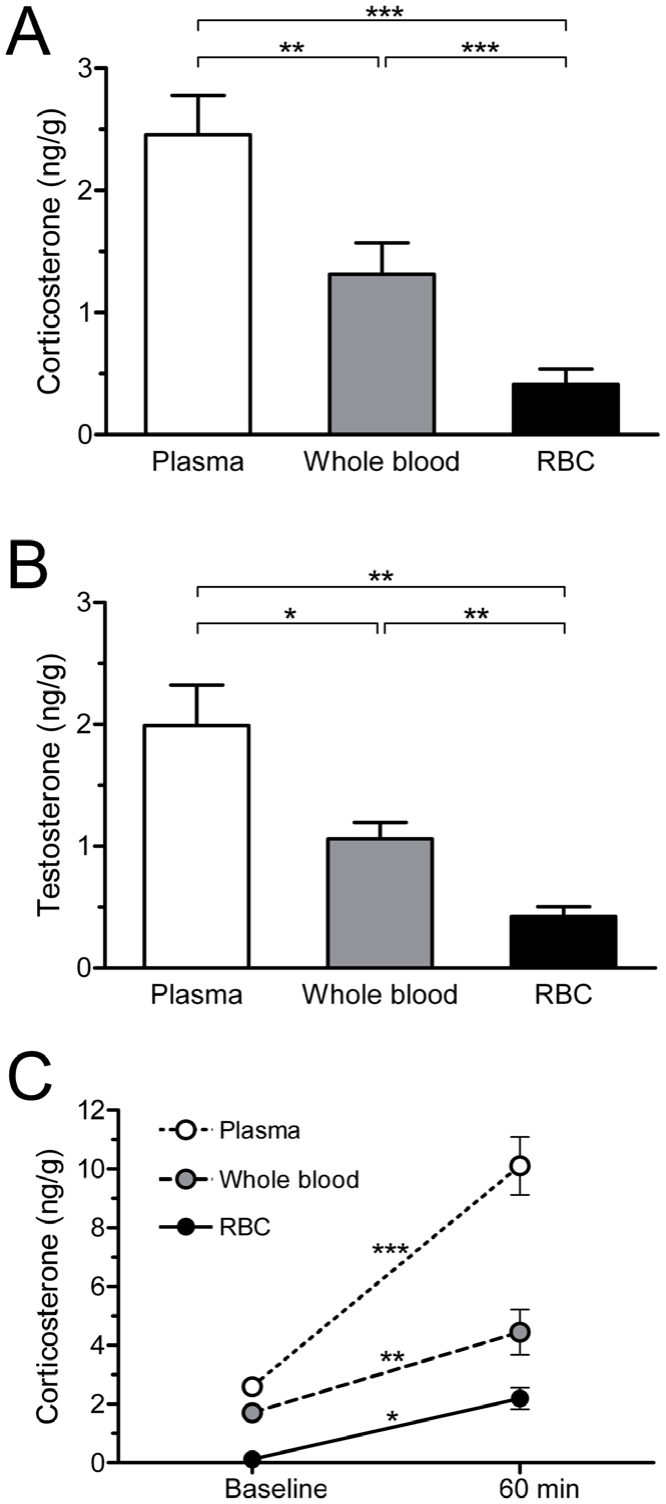
Steroid concentrations in plasma, whole blood, and red blood cells. (**A**) Corticosterone and (**B**) testosterone concentrations in plasma, whole blood, and red blood cells (RBC) of adult male zebra finches (corticosterone n = 12; testosterone n = 5). (**C**) Corticosterone levels in plasma, whole blood, and RBC at baseline (<3 min) and after 60 min restraint stress (baseline n = 6; restraint n = 5). Data are means ± SEM; baseline error bars are too small to be visible. *p≤0.05, **p≤0.01, and ***p≤0.001.

In Study 1A, the distribution of corticosterone in blood compartments was analyzed using a one-way repeated-measures ANOVA with sample type as the within-subjects factor. There was a significant effect of sample type on corticosterone concentrations (F_2,22_ = 32.68, p<0.001, *f* = 0.84) (**Fig. 1A**). Corticosterone concentration was highest in plasma, intermediate in whole blood, and lowest in RBC. *Post hoc* paired t-tests found that each sample type differed from the others (plasma vs. whole blood, t_11_ = 3.685, p = 0.004, d = 1.14; plasma vs. RBC, t_11_ = 8.00, p<0.001, d = 2.44; whole blood vs. RBC, t_11_ = 5.10, p<0.001, d = 1.29). Testosterone in blood compartments was similarly analyzed. There was a significant effect of sample type on T concentrations (F_2,10_ = 18.67, p<0.001, *f* = 0.47) (**Fig. 1B**). Testosterone concentration was highest in plasma, intermediate in whole blood, and lowest in RBC. *Post hoc* paired t-tests found that each sample type differed from the others (plasma vs. whole blood, t_5_ = 3.66, p = 0.02, d = 1.68; plasma vs. RBC, t_5_ = 4.72, p = 0.005, d = 2.65; whole blood vs. RBC, t_5_ = 4.54, p = 0.006, d = 2.09).

In Study 1B, corticosterone distribution across blood compartments at baseline and after 60 min restraint stress was determined using a 2-way mixed-model ANOVA, with sample type as the within-subjects factor and with time (baseline or 60 min) as the between-subjects factor. Corticosterone concentrations differed across sample types (F_2,18_ = 57.75, p<0.001, *f* = 2.53) and between baseline and restraint groups (F_1,9_ = 113.52, p<0.001, *f* = 3.56) (**Fig. 1C**). The sample type × time interaction was also significant (F_2,18_ = 18.48, p<0.001, *f* = 2.33). Analysis of simple main effects (baseline versus 60 min restraint for each sample type) showed that corticosterone concentrations increased in plasma (F_1,9_ = 118.18, p<0.001, d = 4.59), whole blood (F_1,9_ = 15.71, p = 0.003, d = 2.07), and RBC (F_1,9_ = 8.92, p<0.02, d = 2.59). The significant interaction shows that the magnitude of corticosterone increase was greater in plasma than in whole blood or RBC (**Fig. 1C**). Hematocrit was similar at baseline (0.47±0.03) and after 60 min restraint stress (0.50±0.02) (t_7_ = 0.09, p = 0.93, d = 0.06).

### Study 2: Effects of saline perfusion on brain steroid levels

**Figure 2:**
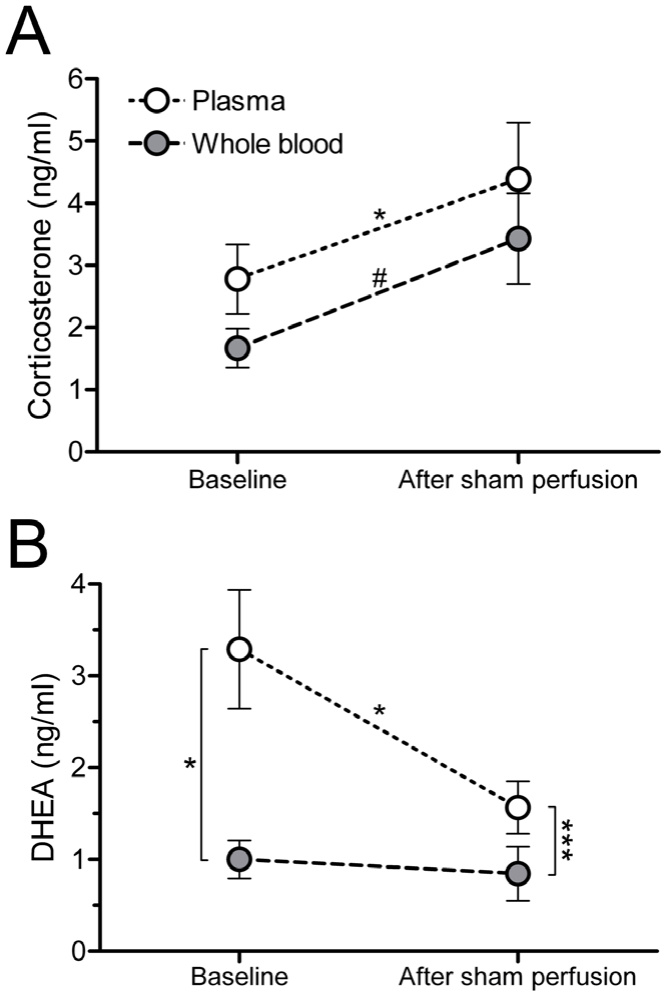
Steroid concentrations in plasma and whole blood before and after sham perfusion. (**A**) Corticosterone and (**B**) DHEA levels in plasma and whole blood at baseline (<3 min) and after sham perfusion (∼11 min) (corticosterone n = 9 baseline, 5 post-sham perfusion; DHEA n = 8 baseline, 3 post-sham perfusion). Baseline blood samples were from the brachial vein, post sham-perfusion blood samples were from the heart. *p≤0.05, ***p≤0.001, #p = 0.06.

In Study 2, steroid concentrations were measured in plasma and whole blood at baseline (both groups) and following sham perfusion (control group only). Blood samples from saline-perfused subjects could only be collected at baseline. Corticosterone levels increased in plasma (t_8_ = 3.33, p = 0.01, d = 1.13) and tended to increase in whole blood (t_4_ = 2.68, p = 0.06, d = 1.17) (**Fig. 2A**). In contrast, DHEA levels decreased in plasma (t_7_ = 2.76, p = 0.03, d = 1.10; **Fig. 2B**) and did not change in whole blood (t_2_ = 0.39, p = 0.74, d = 0.44) (**Fig. 2B**). Testosterone levels were measured in plasma only (insufficient amount of whole blood) and decreased after sham perfusion (1.23±0.22 ng/ml at baseline, 0.95±0.21 ng/ml after sham perfusion) (t_4_ = 3.13, p = 0.04, d = 0.57). There was an insufficient amount of plasma for baseline E_2_ measurement, and cardiac plasma samples (n = 5) were all nondetectable for E2.

**Figure 3:**
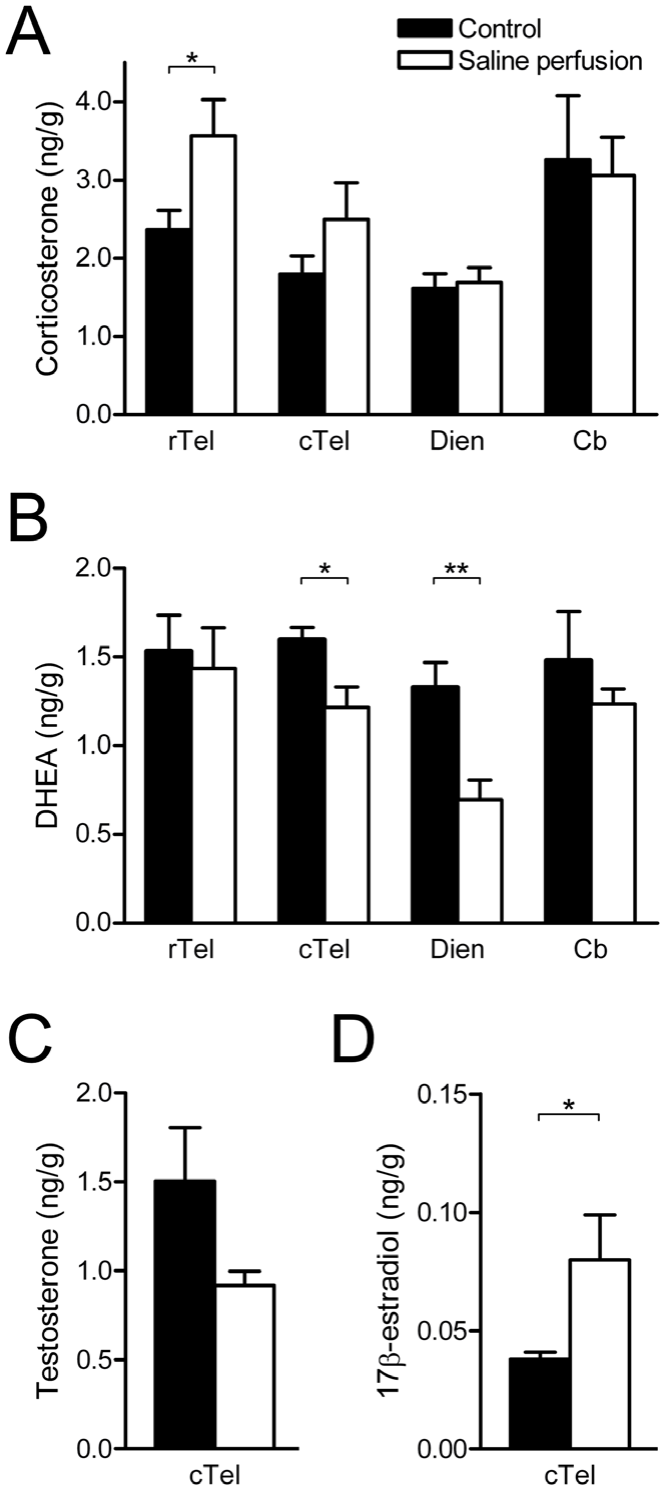
Brain steroid concentrations after saline or sham perfusion. (**A**) Corticosterone, (**B**) DHEA, (**C**) testosterone, and (**D**) 17β-estradiol concentrations were measured in male zebra finch brain regions after saline perfusion or sham perfusion (control). Rostral telencephalon  =  rTel, caudal telencephalon  =  cTel, diencephalon  =  Dien, cerebellum  =  Cb. *p≤0.05, **p≤0.01.

Next, we examined the effects of saline perfusion on brain steroid concentrations. Surprisingly, saline perfusion increased corticosterone levels in the rostral telencephalon (t_12_ = −2.55, p = 0.03, d = 1.34), but had no effect in the caudal telencephalon (t_12_ = −1.51, p = 0.16, d = 0.79), diencephalon (t_11_ = −0.26, p = 0.80, d = 0.15) or cerebellum (t_10_ = −0.04, p = 0.97, d = 0.12) (**Fig. 3A** **)**. Saline perfusion decreased DHEA levels in caudal telencephalon (t_5.18_ = −2.77, p = 0.04, d = 1.65) and diencephalon (t_11_ = 3.52, p = 0.005, d = 1.89), but had no effect in the rostral telencephalon (t_12_ = 0.30, p = 0.77, d = 0.17) or cerebellum (t_11.7_ = −0.66, p = 0.52, d = 0.41) (**Fig. 3B**). In caudal telencephalon, saline perfusion did not affect T levels (t_12_ = −1.57, p = 0.14, d = 0.90) (**Fig. 3C**), but unexpectedly, saline perfusion significantly increased E_2_ levels (t_12_ = −2.94, p = 0.01, d = 1.36) (**Fig. 3D**). We did not measure T or E_2_ in other regions because there was insufficient tissue.

**Figure 4:**
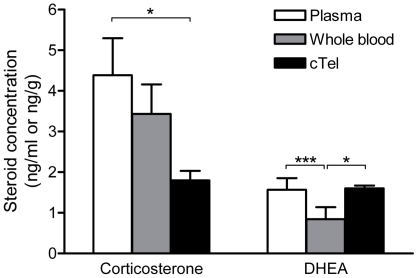
Steroid concentrations in plasma, whole blood, and brain. Corticosterone and DHEA levels in plasma, whole blood, and brain (caudal telencephalon, cTel) of sham-perfused subjects. *p≤0.05, ***p≤0.001.

In sham-perfused subjects, steroid concentrations in plasma, whole blood, and brain at sacrifice were compared within individuals (**Fig. 4**). For brain, the caudal telencephalon was used because all four steroids were measured in this region. Corticosterone concentrations did not significantly differ between plasma and whole blood (t_5_ = 0.69, p = 0.52, d = 0.15). Corticosterone levels were higher in plasma than in brain (t_8_ = −3.02, p = 0.02, d = 1.34), but did not significantly differ between whole blood and brain (t_5_ = −2.02, p = 0.10, d = 1.35). DHEA concentrations were higher in plasma than in whole blood (t_7_ = 6.24, p<0.001, d = 0.76). DHEA levels did not differ between plasma and brain (t_8_ = 0.10, p = 0.92, d = 0.04), but were lower in whole blood than in brain (t_7_ = −2.37, p = 0.0499, d = 1.08). Testosterone levels did not differ significantly between plasma and brain (1.02±0.16 ng/ml in cardiac plasma and 1.30±0.18 ng/ml in brain) (t_6_ = −2.11, p = 0.08, d = 0.59). Note that there was insufficient whole blood for T measurements. E_2_ was non-detectable in all plasma samples, and E_2_ concentrations were significantly lower in plasma than in brain (Wilcoxon test, Z = 2.02, p = 0.04). There was insufficient whole blood for E_2_ measurements.

## Discussion

In this study, we evaluated common methods for measurement and comparison of steroids in the circulation and in brain. In the circulation, we found that corticosterone, T, and DHEA concentrations were lower in whole blood than in plasma. Moreover, restraint stress for 60 min had a larger effect on absolute corticosterone levels in plasma than in whole blood (although the relative increases were similar). These results suggest that use of whole blood samples is beneficial when comparing circulating against tissue steroid levels. In brain tissue, we found that saline perfusion did not result in a global decrease of brain steroid concentrations, indicating that blood contamination does not affect measurement of brain steroid levels. Indeed, saline perfusion had region-specific and steroid-specific effects on brain steroid concentrations, elevating corticosterone levels in rostral telencephalon, decreasing DHEA levels in caudal telencephalon and diencephalon, and increasing E_2_ levels in caudal telencephalon.

### Steroid distribution in blood

In Study 1, corticosterone and T levels were high in plasma, intermediate in whole blood, and low in RBC. Thus, steroid levels in plasma and whole blood can differ, and plasma levels can overestimate circulating levels in whole blood. Similarly, in humans, most cortisol is found in the plasma, bound to CBG [9]. This issue has been well-studied with regard to glucose, which is also present at higher levels in plasma than whole blood [33]. This issue is particularly important for studies comparing circulating and local levels of steroids, because the difference between circulating and local steroid concentrations could be miscalculated. Thus, future studies asking this particular question could benefit from including whole blood samples to estimate circulating steroid levels, especially for comparison with local steroid levels in brain and other organs. Brain regions with relatively modest levels of local steroid production might not be detected if compared only against plasma (or serum).

In Study 1B, restraint stress for 60 min caused a greater increase of absolute corticosterone levels in plasma than in whole blood and RBC (relative increases were similar). This was the opposite of what we had expected. Plasma CBG levels decrease after 60 min of restraint in this species [15], so we expected the rise in corticosterone to be highest, proportionally, in RBC (whose steroid binding capacity would not decrease like plasma CBG). Our findings suggest that even though plasma CBG levels decrease after 60 min, there is still an excess of plasma binding sites, allowing most of the corticosterone to remain in the plasma. This is consistent with a previous study showing a large excess of CBG binding sites in male white-crowned sparrow plasma [16]. Interestingly, the case appears to be opposite in humans, in which the rise in cortisol is greater in RBC than plasma [9]. This possible species difference warrants further investigation.

In Study 2, corticosterone levels increased in plasma and tended to increase in whole blood after sham perfusion (∼11 min after initial disturbance). Plasma and whole blood levels did not significantly differ, in contrast to Study 1, although the pattern appears to be similar. DHEA levels decreased in plasma but did not change in whole blood after sham perfusion. Taken together with Study 1B, these data suggest that stressors can have differential effects on steroid levels in plasma and whole blood. In general, changes in steroid concentrations seem more pronounced in plasma than in whole blood.

### Steroid concentrations in brain

If blood contamination greatly affected brain steroid measurement, then saline perfusion would have caused a global decrease in all brain steroid levels. This was clearly not the case. Saline perfusion changed brain steroid concentrations in a steroid-specific and region-specific manner. These effects were rapid: it took ∼4 min to completely expel the 10 ml of saline from the syringe. Of the four brain areas collected, corticosterone was only affected in the rostral telencephalon, where it was ∼50% higher in saline-perfused subjects than in controls. One possible explanation is that cells in the rostral telencephalon rapidly synthesize corticosterone in response to saline perfusion. The absence of oxygen and glucose induced by the saline perfusion could be a physiological stressor, similar to ischemia. Corticosterone is synthesized in rat brain [5], but there is little evidence for glucocorticoid synthesis in songbird brain, except in molting song sparrows [34]. The present data strongly suggest local synthesis of corticosterone in the adult zebra finch forebrain and also suggest that such local glucocorticoid synthesis can be very rapidly upregulated. The function is unclear, because GCs exacerbate post-ischemic neural injury in adult rodents [35], [36], although GC pretreatment has beneficial effects in neonatal rats [37]. Alternatively, the increase in corticosterone might allow increased aldosterone production in brain. Aldosterone synthesis in the rat brain increases with low salt intake [38], [39], and if our saline was even slightly hypoosmotic, aldosterone (and therefore corticosterone) production might be induced.

Saline perfusion decreased DHEA concentrations by ∼25% in caudal telencephalon and by ∼50% in diencephalon, while saline perfusion increased E_2_ concentrations by ∼100% in caudal telencephalon. Similarly, in breeding song sparrows, restraint stress decreases DHEA levels in jugular plasma, which is enriched with neural steroids [34]. In the caudal telencephalon, the decrease in DHEA levels and increase in E_2_ levels suggest the conversion of DHEA into T and then E_2_ within this region. Caudal telencephalon contains high levels of 3β-HSD [40] and aromatase [41], enzymes that catalyze DHEA conversion to androgens and estrogens, respectively. Activity of these enzymes is rapidly regulated in avian brain [40], [42]–[46]. Similarly, in rats, local E_2_ production increases in response to ischemic stroke [47], and E_2_ has neuroprotective effects in the context of ischemia [48]. Ischemic stroke and saline perfusion, both of which reduce blood supply to neural tissue, may have similar effects on local E_2_ levels by upregulating 3β-HSD and/or aromatase activities.

The observed changes in brain steroid levels after saline perfusion show that saline perfusion has its own direct effects on the brain. These effects are potentially problematic, especially when perfusing prior to analysis of steroidogenic enzymes, steroids, or their downstream signaling cascades (e.g., pERK). Where possible, saline perfusion may be best avoided altogether. When perfusion is deemed necessary, e.g. before fixation for immunocytochemistry, the unwanted effects might be minimized by rapid completion of perfusion and the use of oxygenated artificial cerebrospinal fluid. Non-perfused subjects can also be a useful control [10], [11].

The results of our saline perfusion differ from previous studies in rats [10] and mice [11], in which E_2_ and corticosterone levels in brain were similar in non-perfused and saline-perfused subjects (note that these studies did not include a sham perfusion). Compared to rodents, the songbird brain may have more robust upregulation of steroidogenic enzyme activity during saline perfusion. Additionally, this study compared saline-perfused subjects with sham-perfused subjects.

To estimate the amount of blood removed with perfusion, we attempted to indirectly quantify blood volume in brain by measuring hemoglobin in brain via the cyanmethemoglobin method [49], which has been validated for zebra finch blood [50]. Our preliminary data suggest that blood is only ∼1% of zebra finch brain (v/w). This is similar to ∼0.5% in rats [51] and ∼2% in humans [52].

### Comparing steroid levels in plasma, whole blood, and brain

In the sham-perfused subjects of Study 2, corticosterone levels in whole blood did not differ significantly from those in caudal telencephalon, but corticosterone levels in plasma were higher than those in brain. DHEA levels were similar in plasma and caudal telencephalon, but lower in whole blood than in brain. These data demonstrate that using plasma can overestimate circulating steroid concentrations to a degree that qualitatively affects comparisons with brain steroid levels and alters interpretation of the results. These data do not provide any clear indication about local corticosterone production in caudal telencephalon, but do suggest local DHEA production in caudal telencephalon. High E_2_ levels in brain relative to plasma also suggest local E_2_ production (as in [27]); this difference is robust enough to be detectable, despite using plasma to estimate circulating E_2_ levels. These data show that in studies comparing tissue and circulating steroid levels, whole blood samples should be included as a measure of the circulating steroid levels. Steroid levels in whole blood more clearly reflect what the tissues are exposed to. Also, since steroid levels in whole blood are lower than in plasma, this might improve the ability to detect moderate levels of local steroid synthesis.

In both blood and tissue, we measured total steroid levels, and we cannot address the issue of steroid bioavailability. In zebra finches, 90 to 95% of plasma corticosterone is bound to CBG, and bound corticosterone is considered to have little or no access to tissues [15]. T also binds to CBG [16]. RBC-associated steroids may thus have greater access to tissues than plasma steroids. In rats, brain uptake of corticosterone and T is higher from RBC than from plasma [53].

### Conclusions

First, corticosterone, T and DHEA levels are significantly higher in plasma than in whole blood. Critically, our findings suggest that whole blood samples are useful to assess circulating steroid levels, especially if circulating levels will be compared with local tissue levels. Second, the data suggest that saline perfusion to remove blood contamination is not necessary prior to measurement of brain steroid levels. In fact, saline perfusion has complex region- and steroid-specific effects of its own, perhaps by inducing ischemia. These effects of saline perfusion will generally be undesirable when studying brain steroids or their non-genomic signaling pathways. If exsanguination is necessary, then rapid perfusion with oxygenated artificial cerebrospinal fluid may minimize changes in brain steroid levels.

## References

[1] Schmidt KL, Pradhan DS, Shah AH, Charlier TD, Chin EH (2008). Neurosteroids, immunosteroids, and the Balkanization of endocrinology.. Gen Comp Endocr.

[2] Schmidt KL, Soma KK (2008). Cortisol and corticosterone in the songbird immune and nervous systems: local vs. systemic levels during development.. Am J Physiol-Reg I.

[3] Vacchio MS, Papadopoulos V, Ashwell JD (1994). Steroid production in the thymus: implications for thymocyte selection.. J Exp Med.

[4] Ito N, Ito T, Kromminga A, Bettermann A, Takigawa M (2005). Human hair follicles display a functional equivalent of the hypothalamic-pituitary-adrenal axis and synthesize cortisol.. FASEB J.

[5] Gomez-Sanchez CE, Zhou MY, Cozza EN, Morita H, Foecking MF (1997). Aldosterone biosynthesis in the rat brain.. Endocrinology.

[6] Balthazart J, Ball GF (2006). Is brain estradiol a hormone or a neurotransmitter?. Trends Neurosci.

[7] Do Rego JL, Seong JY, Burel D, Leprince J, Luu-The V (2009). Neurosteroid biosynthesis: enzymatic pathways and neuroendocrine regulation by neurotransmitters and neuropeptides.. Front Neuroendocrin.

[8] Hojo Y, Higo S, Ishii H, Ooishi Y, Mukai H (2009). Comparison between hippocampus-synthesized and circulation-derived sex steroids in the hippocampus.. Endocrinology.

[9] Hiramatsu R, Nisula BC (1987). Erythrocyte-associated cortisol: measurement, kinetics of dissociation, and potential physiological significance.. J Clin Endocr Metab.

[10] Amateau SK, Alt JJ, Stamps CL, McCarthy MM (2004). Brain estradiol content in newborn rats: sex differences, regional heterogeneity, and possible de novo synthesis by the female telencephalon.. Endocrinology.

[11] Little HJ, Croft AP, O'Callaghan MJ, Brooks SP, Wang G (2008). Selective increases in regional brain glucocorticoid: a novel effect of chronic alcohol.. Neuroscience.

[12] Goodson JL, Saldanha CJ, Hahn TP, Soma KK (2005). Recent advances in behavioral neuroendocrinology: Insights from studies on birds.. Horm Behav.

[13] Remage-Healey L, London SE, Schlinger BA (2010). Birdsong and the neural production of steroids.. J Chem Neuroanat.

[14] Newman AEM, Soma KK (2009). Corticosterone and dehydroepiandrosterone in songbird plasma and brain: effects of season and acute stress.. Eur J Neurosci.

[15] Breuner C, Lynn S, Julian G, Cornelius J, Heidinger B (2006). Plasma-binding globulins and acute stress response.. Horm Metab Res.

[16] Charlier TD, Underhill C, Hammond GL, Soma KK (2009). Effects of aggressive encounters on plasma corticosteroid-binding globulin and its ligands in white-crowned sparrows.. Horm Behav.

[17] Hiramatsu R (1983). Determination of cortisol concentration in human erythrocytes.. Clin Chim Acta.

[18] Romero LM, Soma KK, Wingfield JC (1998). Hypothalamic-pituitary-adrenal axis changes allow seasonal modulation of corticosterone in a bird.. Am J Physiol-Reg I.

[19] Roberts JR (1998). The effect of acute or chronic administration of prolactin on renal function in chickens.. J Comp Physiol B.

[20] Schmidt KL, Malisch JL, Breuner CW, Soma KK (2010). Corticosterone and cortisol binding sites in plasma, immune organs and brain of developing zebra finches: Intracellular and membrane-associated receptors.. Brain Behav Immun.

[21] Newman AEM, Chin EH, Schmidt KL, Bond L, Wynne-Edwards KE (2008). Analysis of steroids in songbird plasma and brain by coupling solid phase extraction to radioimmunoassay.. Gen Comp Endocr.

[22] Bélanger A, Couture J, Caron S, Roy R (1990). Determination of Nonconjugated and Conjugated Steroid Levels in Plasma and Prostate after Separation on C-18 Columns.. Ann NY Acad Sci.

[23] Brummelte S, Schmidt KL, Taves MD, Soma KK, Galea LA (2010). Elevated corticosterone levels in stomach milk, serum, and brain of male and female offspring after maternal corticosterone treatment.. Dev Neurobiol.

[24] Washburn BE, Morris DL, Millspaugh JJ, Faaborg J, Schulz JH (2002). Using a commercially available radioimmunoassay to quantify corticosterone in avian plasma.. Condor.

[25] Granger DA, Schwartz EB, Booth A, Curran M, Zakaria D (1999). Assessing dehydroepiandrosterone in saliva: a simple radioimmunoassay for use in studies of children, adolescents and adults.. Psychoneuroendocrinology.

[26] Granger DA, Schwartz EB, Booth A, Arentz M (1999). Salivary testosterone determination in studies of child health and development.. Horm Behav.

[27] Charlier TD, Po KWL, Newman AEM, Shah AH, Saldanha CJ (2010). 17β-Estradiol levels in male zebra finch brain: combining Palkovits punch and an ultrasensitive radioimmunoassay.. Gen Comp Endocr.

[28] Newman AEM, MacDougall-Shackleton SA, An Y-S, Kriengwatana B, Soma KK (2010). Corticosterone and dehydroepiandrosterone have opposing effects on adult neuroplasticity in the avian song control system.. J Comp Neurol.

[29] Schmidt KL, Chin EH, Shah AH, Soma KK (2009). Cortisol and corticosterone in immune organs and brain of European starlings: developmental changes, effects of restraint stress, comparison with zebra finches.. Am J Physiol-Reg I.

[30] Faul F, Erdfelder E, Lang AG, Buchner A (2007). G*Power 3: a flexible statistical power analysis program for the social, behavioral, and biomedical sciences.. Behavior research methods.

[31] Cohen J (1988). Statistical Power Analysis for the Behavioral Sciences. 2nd ed..

[32] Dunlap WP, Cortina JM, Vaslow JB, Burke MJ (1996). Meta-analysis of experiments with matched groups or repeated measures designs.. Psychol Methods.

[33] Holtkamp HC, Verhoef N, Leijnse B (1975). The difference between the glucose concentrations in plasma and whole blood.. Clin Chim Acta.

[34] Newman AEM, Pradhan DS, Soma KK (2008). Dehydroepiandrosterone and corticosterone are regulated by season and acute stress in a wild songbird: jugular versus brachial plasma.. Endocrinology.

[35] Sapolsky RM, Pulsinelli WA (1985). Glucocorticoids potentiate ischemic injury to neurons: therapeutic implications.. Science.

[36] Payne RS, Tseng MT, Schurr A (2003). The glucose paradox of cerebral ischemia: evidence for corticosterone involvement.. Brain Res.

[37] Barks JDE, Post M, Tuor UI (1991). Dexamethasone prevents hypoxic-ischemic brain damage in the neonatal rat.. Pediatr Res.

[38] Ye P, Kenyon C, MacKenzie S, Seckl J, Fraser R (2003). Regulation of aldosterone synthase gene expression in the rat adrenal gland and central nervous system by sodium and angiotensin II.. Endocrinology.

[39] Gomez-Sanchez EP, Ahmad N, Romero DG, Gomez-Sanchez CE (2005). Is aldosterone synthesized within the rat brain?. Am J Physiol-Endoc M.

[40] Soma KK, Alday NA, Hau M, Schlinger BA (2004). Dehydroepiandrosterone metabolism by 3beta-hydroxysteroid dehydrogenase/Delta5-Delta4 isomerase in adult zebra finch brain: sex difference and rapid effect of stress.. Endocrinology.

[41] Saldanha CJ, Tuerk MJ, Kim YH, Fernandes AO, Arnold AP (2000). Distribution and regulation of telencephalic aromatase expression in the zebra finch revealed with a specific antibody.. J Comp Neurol.

[42] Pradhan DS, Lau LYM, Schmidt KL, Soma KK (2010). 3β-HSD in songbird brain: subcellular localization and rapid regulation by estradiol.. J Neurochem.

[43] Pradhan DS, Newman AEM, Wacker DW, Wingfield JC, Schlinger BA (2010). Aggressive interactions rapidly increase androgen synthesis in the brain during the non-breeding season.. Horm Behav.

[44] Pradhan DS, Yu Y, Soma KK (2008). Rapid estrogen regulation of DHEA metabolism in the male and female songbird brain.. J Neurochem.

[45] Remage-healey L, Maidment NT, Schlinger BA (2008). Forebrain steroid levels fluctuate rapidly during social interactions.. Nat Neurosci.

[46] Balthazart J, Baillien M, Ball GF (2001). Rapid and reversible inhibition of brain aromatase activity.. J Neuroendocrinol.

[47] Saleh TM, Connell BJ, Legge C, Cribb AE (2004). Stroke-induced changes in estrogen release and neuronal activity in the parabrachial nucleus of the male rat.. J Stroke Cerebrovasc.

[48] Dubal DB, Shughrue PJ, Wilson ME, Merchenthaler I, Wise PM (1999). Estradiol modulates bcl-2 in cerebral ischemia: a potential role for estrogen receptors.. J Neurosci.

[49] Drabkin DL, Austin JM (1932). Spectrophotometric constants for common hemoglobin derivatives in human, dog and rabbit blood.. J Biol Chem.

[50] Wagner EC, Prevolsek JS, Wynne-Edwards KE, Williams TD (2008). Hematological changes associated with egg production: estrogen dependence and repeatability.. J Exp Biol.

[51] Wang MD, Wahlström G, Bäckström T (1997). The regional brain distribution of the neurosteroids pregnenolone and pregnenolone sulfate following intravenous infusion.. J Steroid Biochem.

[52] Bradbury MWB (1979). The Concept of a Blood-Brain Barrier..

[53] Hiramatsu R, Nisula BC (1991). Uptake of erythrocyte-associated component of blood testosterone and corticosterone to rat brain.. J Steroid Biochem.

